# Fungi-Derived Bioactive Compounds as Potential Therapeutic Agents for Pancreatic Cancer: A Systematic Review

**DOI:** 10.3390/microorganisms12081527

**Published:** 2024-07-25

**Authors:** Francisco Quiñonero, Alba Ortigosa-Palomo, Raul Ortiz, Consolacion Melguizo, Jose Prados

**Affiliations:** 1 Institute of Biopathology and Regenerative Medicine (IBIMER), Center of Biomedical Research (CIBM), University of Granada, 18100 Granada, Spain; fjquinonero@ugr.es (F.Q.); albaortigosa@ugr.es (A.O.-P.); roquesa@ugr.es (R.O.); jcprados@ugr.es (J.P.); 2 Biosanitary Institute of Granada (ibs.GRANADA), SAS-University of Granada, 18014 Granada, Spain; 3 Department of Anatomy and Embryology, Faculty of Medicine, University of Granada, 18071 Granada, Spain

**Keywords:** pancreatic cancer, metabolites, fungi, anticancer effect

## Abstract

Pancreatic cancer (PC) is one of the tumors with the lowest 5-year survival rate worldwide due to late diagnosis and lack of effective therapy. Because of this, it is necessary to discover new ways of treatment to increase the quality of life of patients. In this context, the secondary metabolites of several fungi have been shown as a possible therapeutic strategy in several types of cancer, such as colorectal cancer, being able to trigger their action through the induction of apoptosis. The objective was to perform a systematic review process to analyze the studies carried out during the last ten years using secondary metabolites derived from fungi as antitumor treatment against PC. After the search process in three databases (PubMed, SCOPUS, and Web of Science) a total of 199 articles were found, with 27 articles finally being included after screening. The results extracted from this systematic review process made it possible to determine the existence of bioactive compounds extracted from fungi that have been effective in in vitro and in vivo conditions and that may be applicable as a possible therapy to avoid drug resistance in PC, one of the major problems of this disease.

## 1. Introduction

Pancreatic cancer is one of the most lethal tumor types today, having undergone a 2-fold increase in its incidence worldwide over the past 20 years (from 196,000 cases in 1990 to 495,000 in 2020) and being projected to become the second leading cause of cancer death in the United States by 2030 [1]. One of the main causes of the high mortality rate of this type of cancer is its late diagnosis, which results in more than 50% of patients being diagnosed in advanced stages, generally metastatic [2,3]. Risk factors predisposing to its development include smoking, alcohol consumption, diabetes mellitus, and overweight, among other things. On the other hand, there are certain genetic factors that predispose individuals to its development, such as mutations in genes involved in the repair of DNA damage (*BRCA1*, *BRCA2*, *PALB2*, *ATM*) or in others involved in the regulation of the cell cycle (*CDKN2A*) [3]. 

Regarding treatment, currently the only way to cure it completely is by surgery in patients in early stages. In addition, neoadjuvant chemotherapy is usually used to avoid the possible appearance of metastatic lesions and to try to reduce the size of the tumor to be excised. In metastatic patients, chemotherapy is usually the most used route of treatment, mainly using FOLFIRINOX, Gemcitabine, or nab-Paclitaxel regimen [4]. Despite the use of all these drugs, pancreatic cancer is highly resistant to chemotherapy due to the existence of a dense extracellular matrix, a hypoxic tumor microenvironment, the presence of tumor stem cells, and the deregulation of several molecular pathways, resulting in therapy often being ineffective in its treatment [5]. For this reason, new targeted therapies have been developed in recent years due to advances in technologies such as sequencing. Thus, PARP inhibitors such as Olaparib are being used in patients with mutations in DNA repair pathways (such as BRCA), and therapy is also focusing on inhibiting other proteins involved in tumor progression, such as KRAS, ATM, and PI3K, among others [6]. In addition, the new discovery of compounds with antitumor capacity is essential to obtain increasingly effective treatments. 

During recent years, the use of different natural products derived from nature with proven antitumor properties has been extended, being able to exert a cytotoxic effect through several pathways and avoiding the traditional drug resistance phenomena. Thus, some products, such as curcumin or resveratrol, among others, could exert their antitumor action by generating apoptosis, inhibiting metastasis mechanisms, and possessing anti-angiogenic capacities [7]. Among all organisms capable of generating bioactive compounds, fungi represent the largest eukaryotic kingdom, being able to synthetize many secondary metabolites in response to their prey, to ultraviolet radiation, and to their competition against other microorganisms. Then, they are a great source of potential bioactive compounds that have been studied in recent years, both in marine and terrestrial fungi [8,9,10]. The compounds synthesized include alkaloids, coumarins, flavonoids, lignans, saponins, and terpenes, among others. These cannot be synthesized by animals, since they do not have this type of metabolic pathway, but they may be good candidates as antitumor treatment in several types of cancer, such as colorectal or pancreatic cancer [9,11]. Among all genera, *Aspergillus* and *Penicillium* have the highest number of secondary compounds with antitumor activity. Thus, the enzyme with the greatest antitumor capacity (asparaginase) has been isolated from strains of *Aspergillus* (*niger*, *tubingensis*, *terreus*), *Tamaromyces pinophilis*, *Trichoderma viride*, etc. [12]. Therefore, fungal compounds may be an interesting strategy for the discovery of new antitumor drugs to treat PC.

Consequently, the purpose of this systematic review is to identify various compounds derived from fungi that have been used to treat PC over the past decade. The review will analyze the antitumor efficacy of these compounds in both in vitro cellular models and in vivo murine models.

## 2. Materials and Methods

### 2.1. Study Eligibility

The purpose of this study is to identify bioactive compounds extracted from fungi (terrestrial or marine) or chemically synthesized derivatives of these molecules that possess cytotoxic activity against PC tumor cell lines. The systematic review was carried out in accordance with the PRISMA 2020 guidelines [13]. Studies published in the last ten years were selected for the review, thus avoiding the incorporation of older information that may be obsolete.

### 2.2. Inclusion Criteria

The articles included in the systematic review had to be published between December 2013 and December 2023, available in open-access journals and written in English or Spanish to facilitate the reviewers’ understanding. In addition, studies carried out in lines of all subtypes of PC were included, although those carried out in pancreatic ductal adenocarcinoma lines were the most common.

### 2.3. Exclusion Criteria

All articles using fungal-derived active compounds that were not tested in PC cell lines were excluded from this review. In addition, all publications in which the compounds used were of commercial origin were excluded; we confined our search only to studies that obtained them by direct isolation of a fungus or by synthesis of chemical derivatives of these compounds. Finally, we excluded all types of articles that were not original research publications, like reviews, systematic reviews, and conference abstracts, among others.

### 2.4. Data Sources

The PubMed, SCOPUS, and Web of Science (WoS) databases were used for the systematic search of the publications to be included in this systematic review. The search formula varied among them, due to their own characteristics. For PubMed, the following formula was used: ((Pancreatic Neoplasms[MeSH Terms]) OR (pancrea* cancer*[Title/Abstract]) OR (pancrea* neoplasm*[Title/Abstract]) OR (pancrea* tumor*[Title/Abstract])) AND ((Fungi[MeSH Terms]) OR (fung*[Title/Abstract])) AND ((Antineoplastic Agents[MeSH Terms]) OR (Bioactive*[Title/Abstract]) OR (Antitumor*[Title/Abstract]) OR (Anticancer*[Title/Abstract])). 

For the SCOPUS database, it was as follows: (TITLE-ABS (Pancreatic Neoplasms) OR TITLE-ABS (pancrea* cancer) OR TITLE-ABS (pancrea* AND tumor*)) AND (TITLE-ABS (fungi) OR TITLE-ABS (fungus) OR TITLE-ABS (fungal)) AND (TITLE-ABS (bioactive) OR TITLE-ABS (antitumor) OR TITLE-ABS (anticancer) OR TITLE-ABS (antineoplastic)) AND PUBYEAR > 2012 AND PUBYEAR < 2024 AND PUBYEAR > 2012 AND PUBYEAR < 2024 AND (LIMIT-TO (DOCTYPE,”ar”)). 

Finally, the formula used in WoS was as follows: TS = ((pancrea* neoplasm* OR pancrea* cancer* OR pancrea* tumor*) AND (Fungi OR Fungus) AND (bioactive OR antitumor OR anticancer OR antineoplastic)). All bibliographic information extracted from the searches was stored in the bibliography software Mendeley Reference Manager 2.112.0 (Elsevier, Amsterdam, The Netherlands).

### 2.5. Study Selection

The lead author (F.Q.) performed the literature search in the databases. Initially, a comparison was made between databases, locating those articles that were duplicated and discarding them. In addition, the articles were selected by title and abstract, initially checking whether they met the inclusion and exclusion criteria. In a second step, two authors (F.Q. and A.O.-P.) reviewed the full text of the articles, carrying out a quality assessment based on criteria previously established by both authors (Table 1). To make the score, a distinction was made between fundamental aspects for the quality of the article (3 points), aspects important to ensure it (2 points), and complementary aspects (1 point). Articles that obtained a score less than 12 (0–11) were discarded due to low quality, while the rest were classified as medium (12–16) or high quality (17–20). If both authors differed regarding the quality score of a publication, a third reviewer would be introduced to establish the final score.

### 2.6. Data Extraction

Finally, data extraction was carried out from the articles that met the quality criteria. The information retrieved included the specific fungal species from which the compound had been extracted, the name of the extracted compound, information on the solvent used for extraction or the synthesis method, IC_50_ value of the compounds in the pancreatic tumor cell lines, regulation of oncogenic pathways by investigated compounds, the most relevant results extracted from in vitro tests, and the results obtained in in vivo experiments (if they were carried out). 

## 3. Results

### 3.1. Study Description

**Figure 1:**
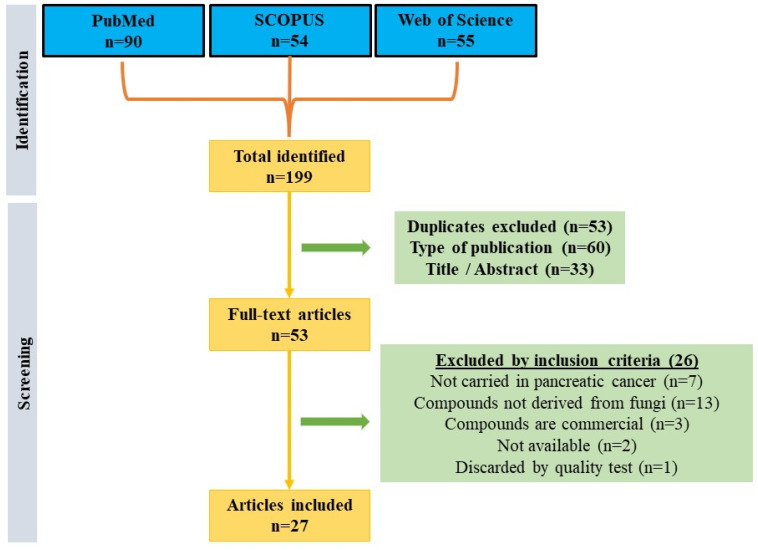
Flow diagram that represents the articles included in the systematic review.

Following a systematic search in the main databases (PubMed, SCOPUS, and WoS), a total of 199 articles were obtained. From there, 53 duplicate articles between the different databases were excluded, 60 publications were excluded because they did not deal with original research, and 33 articles were discarded because of their title/abstract in a primary screening. Subsequently, of the remaining fifty-three articles, seven studies were excluded for not being conducted in pancreatic cancer lines, thirteen for not using fungal-derived compounds, three for using commercial compounds, two for not finding the articles available, and one for not having sufficient quality (score less than 12). Therefore, a total of 27 articles were finally included in this review (Figure 1). It should be noted that of the articles included, only six passed the quality test with a high score, while the rest were classified as of medium quality.

**Figure 2:**
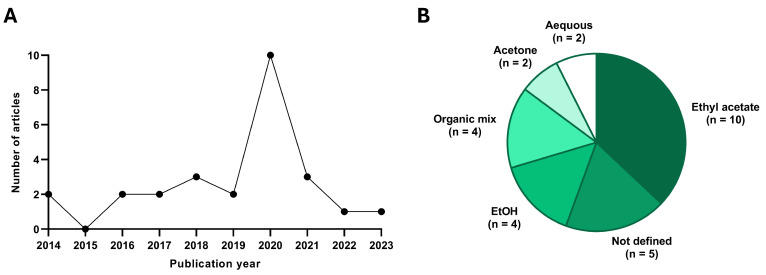
Statistical data extracted from the publications included. (**A**) Quantity of studies published in each year and (**B**) solvent used for extraction from the fungus. *n* indicates the number of studies which used each solvent.

Most of the studies have been carried out in the last 5 years (18/27) (Figure 2), so the discovery of these bioactive molecules is recent. In addition, when analyzing the species of fungi from which they derive, we must highlight that twenty-four out of twenty-seven studies are carried out on compounds derived from the phylum Ascomycota, while the remaining three belong to the phylum Basidiomycota. Another aspect we must emphasize is that the compounds are generally obtained by extraction from the fungus using an organic solvent (ethyl acetate, ethanol, acetone, methanol), followed by isolation of specific fractions by HPLC. In addition, many studies perform tests complementary to cytotoxicity to discover the mechanisms by which the compound performs its effect, some of them even testing the compound in xenograft models generated from pancreatic cancer tumor lines.

Since the compounds are derived from fungi belonging to different phyla, the results will be separated according to these criteria.

### 3.2. Cytotoxic Effect of Bioactive Compounds Derived from Fungi of the Order Eurotiales

The genus with the most active compounds within this order were *Penicillium* and *Aspergillus*. Within the genus *Aspergillus*, the fungus from which most metabolites were derived is *Aspergillus terreus*. Derived from this, Yan et al. were able to isolate several compounds by HPLC, among them one called aspulvinone H. This was able to selectively inhibit the enzyme GOT1, involved in the energy metabolism of the cell. After being tested in pancreatic tumor lines, in colon cancer lines, and in a non-tumor line, it was observed that in the tumor lines SW1990, PANC-1, and AsPC-1, it had an IC_50_ between 6.32 and 10.47 µM, so it had a selective activity against this type of cancer. This inhibition of energy metabolism also caused the generation of intracellular ROS, which ended up generating apoptosis, cell cycle arrest in the S phase, and inhibition of migration capacity in the SW1990 line. In addition, an in vivo experiment was performed in mice induced from the SW1990 line, where it was observed that treatment with the compound at 2.5 and 5 mg/kg was effective in inhibiting tumor growth, observing a depletion in energy pathways in the tumor tissue [14]. Derived from this same fungus, Deng et al. isolated 14 different compounds by HPLC. Of these, three showed cytotoxic activity in PC cells: (−)-asperteretone E, (+)-asperteretone E, and compound 6 (unnamed). These showed an IC_50_ ranging from 1.2 to 15.6 µM in pancreatic adenocarcinoma lines AsPC-1, SW1990, and PANC-1 [11]. On the other hand, Qi et al. were able to isolate by HPLC from *Aspergillus terreus* 12 butenolide derivatives and characterize their efficacy in cell lines of different tumor types. Among them, (+)-3′,3′-di-(dimethylallyl)-butyrolactone II and versicolactone B showed efficacy in the pancreatic tumor line PANC-1, with IC_50_ of 5.3 and 9.4 µM, respectively. In addition to this, the authors wanted to demonstrate the pathways by which they exerted their effect, discovering that both compounds caused a dose-dependent increase in apoptosis, in addition to producing an arrest in the S-phase of the cell cycle [15]. Furthermore, Abdel-Naime et al. were able to isolate two bioactive compounds through organic extraction with acetone from an undetermined strain (*Aspergillus* sp. 18B-15-3): physcion and 2-(2′,3-epoxy-1′,3′,5′-heptatrienyl)-6-hydroxy-5-(3-methyl-2-butenyl) benzaldehyde. Both compounds showed 170- to 505-fold efficacy in PANC-1 cells cultured in low-glucose media versus normal glucose levels, decreasing oxygen consumption and membrane potential in this pancreatic adenocarcinoma line [16]. 

Other authors were able to isolate a compound called secalonic acid F from the fungus *Aspergillus aculeatus* among a total of 296 natural compounds. Although the method for its extraction was not detailed, this compound had low IC_50_ values, making it highly effective in the pancreatic adenocarcinoma lines BxPC-3 and SU86.86 (2.5 and 3 µM, respectively). Immunofluorescence analysis of the genotoxicity marker yH2AX showed that this toxicity was due to the generation of double-stranded DNA damage [17]. Within this same genus, Ye et al. isolated a novel cyclopeptide called clavatustide C produced as a stress response metabolite by *Aspergillus clavatus* C2WU. This was elicited in pancreatic, gastric, colorectal, retinoblastoma, and prostate cancer lines. The results in PC showed an IC_50_ ranging from 30–40 to 20–30 µg/mL after 48 and 72 h of treatment in the PANC-1 cell line. This inhibition of proliferation was caused by the induction of a G1/S phase arrest, observing how these types of compounds deregulated the expression of several genes involved in this transition, such as *GSPT2* and *USP2*, among others. Finally, the authors discovered that this antitumor activity was linked to the inhibition of cyclin E2 (*CCNE2*) and the overexpression of a tumor suppressor gene (*CYLD*), both involved in cell cycle regulation [18]. Finalizing the results obtained in organisms of this genus, the secondary metabolite heptelidic acid derived from the fungus *Aspergillus oryzae* was isolated using HPLC in its supernatant. The results showed that the compound generated an inhibition of cell proliferation at doses of 1 and 10 µg/mL in PC lines SUIT-2, MIA PaCa-2, and PANC-1. In addition, they conducted an in vivo experiment using a xenograft model of SUIT-2, where they observed a decrease in tumor growth induced by the induction of apoptosis, the deregulation of proteins involved in cell cycle continuation, and a deregulation of the p38/MAPK signaling pathway [19]. 

In the Penicillium genus, Shi et al. identified a citrinin derivative (possessing pharmacological, anti-inflammatory, and antitumor activity) called dicitrinone G. In this case, the authors performed an ethyl acetate extraction of the whole fungus and performed both in vitro and in vivo tests. Within the in vitro tests, they performed an angiogenesis experiment using conditioned media derived from BxPC-3 cells treated with dicitrinone G in HUVEC endothelial cells. Their results showed a decrease in angiogenic capacity under hypoxic, tumor sinus-like conditions through negative regulation of interleukin IL-18. These results were verified in xenograft models induced from the BxPC-3 line, where it was observed that the compound produced an inhibition in tumor growth of more than 50% compared to untreated controls (in dose ranges between 0.25 and 1 mg/kg), obtaining an efficacy like the use of a chemotherapeutic such as 5-FU. In addition, a decrease in the expression in tumors of the CD31 marker, linked to metastatic processes, was observed in all cases [20]. In addition, Koul et al. isolated several bioactive compounds from the endophytic fungus *Penicillium pinophilum*, among which skyrin and dicatenarin stood out for their bioactive activity. Both compounds were tested in several cell lines of different types of cancer (lung, colon, breast, prostate, ovarian), although they highlight their antitumor activity against the pancreatic adenocarcinoma line MIA PaCa-2, with IC_50_ of 27 and 12 µg/mL, respectively. Both compounds showed their activity through ROS-mediated induction of apoptosis. Additionally, they showed an inhibitory capacity of clonogenicity in the cell line at their highest concentrations (20 and 50 µg/mL in dicatenarin and skyrin), so they were postulated as compounds with an interesting antitumor activity [21]. 

**Figure 3:**
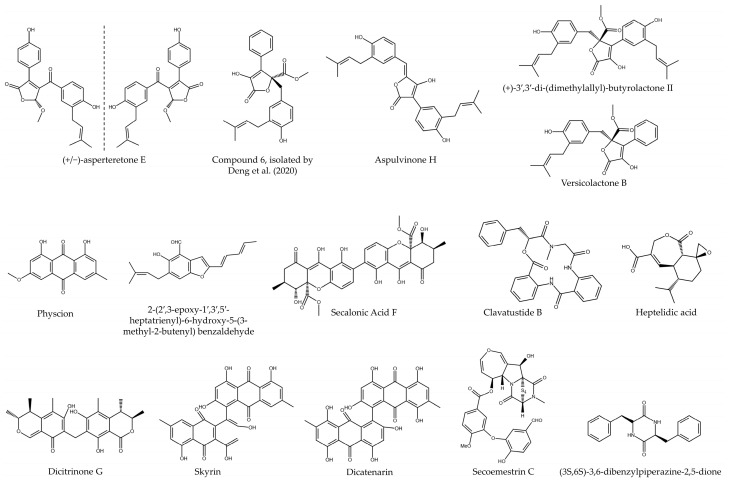
Chemical structure of bioactive compounds isolated from fungi of the order Eurotiales (Compund **6**, isolated by ref. [11]).

Among other genera that produce secondary metabolites of interest, a molecule called secoemestrin C (sec C) has been identified from *Emericella striata* and *Emericella quadrilineata* species. This metabolite was tested in several PC lines, such as AsPC-1, BxPC-3, MIA PaCa-2, PANC-1, SU86.86, and SW1990, and was also tested in comparison with the drug Gemcitabine. The results showed that the IC_50_ at 48 h was in the range of 1 to 4 µM for all these lines, with this drug being especially effective in those lines possessing resistance to Gemcitabine (such as MIA PaCa-2, PANC-1, SU86.86, and SW1990), while its efficacy was similar in those sensitive ones. In addition, sec C was shown to be effective in inhibiting clonogenic capacity in BxPC-3 and MIA PaCa-2 lines, this antitumor activity being mediated by an inhibition of the ER-YAP1 pathway, linked to increased survival in patients and increased chemosensitivity. Finally, the authors demonstrated that treatment with this drug generated death by apoptosis, observed by Western blot through PARP and caspase-3 cleavage [22]. Finally, an investigation carried out to discover metabolites from a *Paecilomyces formous* 17D47-2 strain resulted in the isolation of (3S,6S)-3,6-dibenzylpiperazine-2,5-dione, which possessed an IC_50_ of 28 µM in glucose-deficient media, while it increased to more than 1 mM when PANC-1 cells were grown in media with normal amounts of glucose. This effect was caused because the compound was able to increase the rate of oxygen consumption in the cells, resulting in glucose deficiency leading to cell death [23] (Table 2 and Figure 3). 

### 3.3. Cytotoxic Effect of Bioactive Compounds Derived from Fungi of the Order Hypocreales

Regarding different compounds derived from the order Hypocreales, a study described a new compound with antitumor activity in the PANC-1 line called beauvericin isolated from the genus *Isaria* sp. In this case, four compounds were isolated by HPLC, showing that beauvericin was the only one with antitumor activity, showing an IC_50_ of 4.8 µM. In addition, the compound was able to dose-dependently inhibit cell migration and produce a negative regulation of genes involved in epithelial–mesenchymal transition, such as N-cadherin and Snail [24].

Another compound that showed antitumor activity in these studies was cordycepin, isolated by the group of Li et al. [25] from the fungus *Cordyceps militaris*. In this case, the method of extraction of the compound was not detailed, although its antitumor capacity was tested in several PC cell lines. The results showed an IC_50_ of 38.85, 72.99, 150.1, 213.1, and 349.3 µM in BxPC-3, CFPAC-1, AsPC-1, PANC-1, and SW1990 lines, thus showing significant differences in sensitivity to it among different cell lines. Additionally, the authors performed several in vitro assays on the BxPC-3 line, observing a decrease in clonogenicity and cell migration after treatment. These effects were accomplished by a dose-dependent induction of apoptosis, characterized by annexin V-IP and identification of caspases 3 and 9 cleavages by Western blot. In addition, there was an increase in the expression of pro-apoptotic genes such as *BAX* and *FasL*. Another effect induced by this compound was an S-phase cell cycle arrest, decreasing the expression of cyclin A2 (essential for cycle progression), among others. When we tried to identify the target on which the compound acted, we identified its binding to FGFR2, being able to block the Ras/ErK molecular pathway. Finally, the authors conducted an in vivo experiment using xenograft models of BxPC-3, showing an inhibition of tumor growth through the deregulation of the FGFR/RAS/ERK pathway and a negative regulation of Ki67 expression, a proliferation marker [25]. Another investigation performed by Belur et al. identified a novel lectin with binding capacity to fucosylated glycans expressed in hepatocellular carcinoma and pancreatic cancer in the *Cephalosporium curvulum* strain. The compound, called CSL, was compared to other lectins called LCA and AOL. All three lectins showed antitumor effects in the PANC-1 and HEPG2 lines, demonstrating an IC_50_ value for CSL in the pancreatic line between 2.5 and 5 µg/mL. Added to this, it was shown that CSL was able to induce this effect through the production of apoptosis (through cytometry and Western blot) and the induction of ROS in the HEPG2 hepatocarcinoma cell line [26]. 

Meanwhile, Tang et al. performed the isolation of a cytotoxic compound called epidithiodiketopiperazine DC1149B from *Trichoderma lixii* strain 15G49-1 using HPLC. In this case, the cytotoxic effect shown was increased in low-glucose culture media, resulting in an IC_50_ of 0.02 µM in the PANC-1 line in glucose-deficient media and 710 mM in media with normal glucose levels. The molecular pathway through which it takes effect is based on stress-mediated inhibition of ER signaling, coupled with inhibition of electron transport in the mitochondrial chain [27]. Another fungus that possesses secondary metabolites of interest is *Purpureocillium lilacinum* 40-H-28, where two compounds with biological activity were isolated by HPLC: leucinostatin A and leucinostatin Y. After in vitro tests in the PANC-1 cell line, it was observed that they had an IC_50_ of 0.05 and 4.1 µg/mL, respectively, with superior efficacy in glucose-deficient media. This behavior was similar in other pancreatic adenocarcinoma lines such as BxPC-3, PSN-1, and PK-8 [28]. 

**Figure 4:**
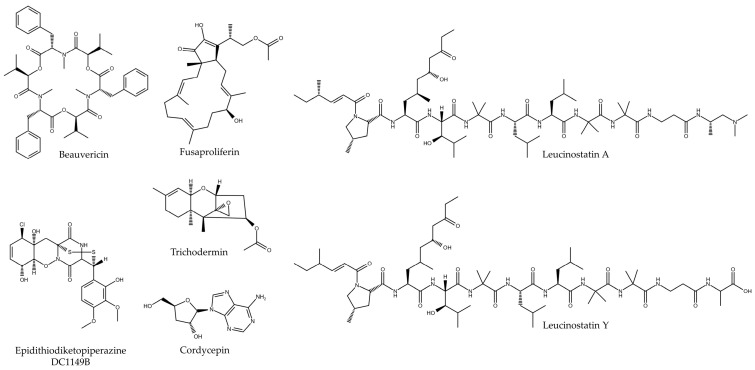
Chemical structure of bioactive compounds isolated from fungi of the order Hypocreales.

Another bioactive compound extracted from the fungus *Fusarium solani* was fusaproliferin, with cytotoxic activity in PC. After its isolation by organic extraction with ethylacetate and isolation by HPLC, a compound was obtained that had an IC_50_ of 0.13 and 0.76 µM in the MIA PaCa-2 and BxPC-3 lines, improving this value compared to the use of the chemotherapeutic Gemcitabine that obtained values of 7.6 and 2.2 µM in the same cell lines [29]. Chien et al. were able to isolate and characterize a compound derived from the fungus *Nalanthamala psidii* called trichodermin. Initially, they tested its cytotoxic potential in three PC cell lines and one non-tumorigenic line, obtaining IC_50_ values of 0.8, 1.2, 1.4, and 18.8 µM in the MIA PaCa-2, BxPC-3, HPAC, and hTERT-HPNE (non-tumorigenic) lines, respectively, in addition to producing a decrease in its clonogenicity. Its cytotoxic effect was exerted through the induction of apoptosis, with its administration producing a cleavage of caspase-9, caspase-3, and PARP. In addition, certain pro-apoptotic proteins such as Bcl-2 and Bad were overexpressed, while the expression of Bcl-xL and Bax (anti-apoptotic) was decreased. In addition, this compound produced a G2/M phase arrest of the cell cycle, which was also verified by increasing the expression of certain cyclins, such as A2 or B1, by Western blot. Finally, it was also shown to be capable of producing DNA damage, being able to activate p53 and generating cell death in p53-mutated cells. In addition to this, an in vivo assay was performed in MIA PaCa-2 xenograft models where a great decrease in tumor growth was observed, obtaining final tumor weights in treatments with 1 and 5 mg/kg of trichodermin similar to the use of gemcitabine at 40 mg/kg doses, without producing any systemic toxicity in the mice. Western blot analysis of tumor tissues showed an induction of apoptosis by cleavage of caspase-3 and PARP, as well as activation of the JNK molecular pathway through its phosphorylation. Immunohistochemical analysis determined an increased phosphorylation of H2AX in tumor tissues treated with the drugs, symptomatic of the production of DNA double-strand damage [30] (Table 3 and Figure 4).

### 3.4. Cytotoxic Effect of Bioactive Compounds Derived from Fungi of Other Orders

Among the active compounds that do not belong to the orders listed above, Fares et al. succeeded in isolating a molecule called striatal C from the fungus *Cyathus striatus* by extraction and HPLC. Cytotoxicity results showed antitumor effectiveness against the PC cell lines, with IC_50_ in the range of 2.5 and 5 (HPAF-II) and close to 2.5 (PL45) µg/mL. Furthermore, the antitumor effect was mediated by the induction of apoptosis, showing increased TUNEL labeling after the cells were treated with the compound [31]. On the other hand, Ghosh and Sanyal created a complete extract by ethanolic extraction through the fungus *Calocybe indica*. This extract was tested in two pancreatic adenocarcinoma lines, obtaining IC_50_ values of 245 and 332 µg/mL in the PANC-1 and MIA PaCa-2 lines. In addition, after analyzing by Western blot the expression of apoptosis markers, it was concluded that the cytotoxic effect was being exerted by this route. Additionally, this extract showed efficacy in decreasing cell migration capacity in the PANC-1 cell line [32]. Finally, Matsushita et al. carried out an aqueous extract from the fungus *Agaricus blazei* Murrill. Its antitumor efficacy was tested in three PC tumor lines (MIA PaCa-2, PCI-35, and PK-38) and one non-tumor line (HPDE), showing a decrease in cell proliferation after treatment. Additionally, an induction of apoptosis was observed through the TUNEL assay and cell cycle arrest in the GO/G1 phase in the PCI-35 and PK-8 cell lines [33].

On the other hand, Zhang et al. were able to isolate a diterpenoid from a fungus of the genus Eutypella called libertellenone H (LH). The authors tested its effectiveness on several PC lines, with IC50 of 0.67, 2.78, 3.21, and 5.53 µM in SW1990, AsPC-1, PANC-1, and BxPC-3, respectively. Added to this, this value was shown to be higher in the non-tumor line HPEDC. By testing the mechanism by which it performed this activity, it was observed that in the SW1990 and PANC-1 lines, it was able to induce apoptosis in a dose-dependent manner. This apoptosis was caused by an increase in intracellular ROS production, reversing the toxicity caused by administration of the antioxidant N-acetylcysteine. Further analysis showed that LH was able to deregulate the Trx system of the cell, thus producing an oxidative imbalance [34].

Another fungus, in this case belonging to another genus (*Pestalotiopsis neglecta* LK29), was able to synthesize nine secondary metabolites, two of which had cytotoxicity against PC. In this case, the compounds pestalone C and pestalone E were extracted using a 1:1 ethyl acetate–methanol mixture and subsequently isolated by HPLC. The results obtained showed that they possessed an IC_50_ of 7.6 and 7.2 µM in the PANC-1 line, this toxicity being induced through apoptosis (shown by cleavage of PARP and caspase-3 by Western blot). When the pathway by which these metabolites acted was analyzed, it was observed that they produced a decrease in the activation of the MEK/ERK pathway, allowing a lower clonogenicity in the treated cells [35].

Pérez-Bonilla et al. carried out the isolation of hormonemate derivatives from the endophytic fungus *Dothiora* sp. Among all these, a compound named hormonetame E was isolated and tested in hepatocellular carcinoma lines, breast cancer, and in the pancreatic MIA PaCa-2 line. In the latter, it obtained an IC_50_ of 36.4 µM, higher than the drug with which it was compared (Doxorubicin, 1.9 µM), but with an interesting antitumor activity [36].

**Figure 5:**
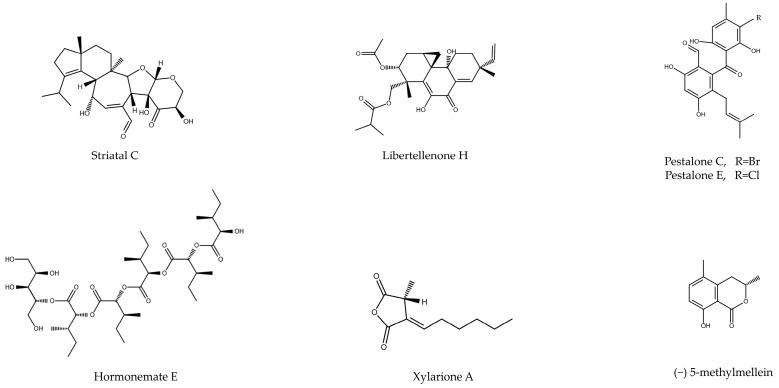
Chemical structure of bioactive compounds isolated from fungi of different orders (e.g., *Agaricales*, *Xylariales*).

Other interesting secondary metabolites were isolated from the fungus *Xylaria psidii*. Arora et al. performed an extraction using ethylacetate from it and isolated two bioactive compounds by HPLC named xylarione A and (−) 5-methylmellein. After being tested in the MIA PaCa-2 line, IC_50_ values of 16 and 19 µM were obtained. In addition, the authors showed that the compounds induced a G0/G1 phase arrest of the cell cycle, also decreasing the cell membrane potential [37]. Although most of the studies seen previously performed isolation of bioactive compounds, Lee et al. obtained two complete extracts using ethyl acetate (EAE) and ethanol (EE) from the mushroom *Antrodia camphorata.* The cytotoxicity results obtained in the BxPC-3 line showed IC_50_ values of 2.49 and 2.36 µg/mL, respectively. Additionally, it was shown that both extracts inhibited cell migration more effectively than the drug Gemcitabine. The cytotoxic effect was exerted through the induction of apoptosis, promoting the expression of pro-apoptotic proteins such as BAX, or through the activation of the caspase pathway, with the induction of cleavages in caspase-3, 9 and PARP being observed by Western blot [38]. The same approach was carried out in a study concerning the fungus *Colletotrichum gleosporioides,* where Sheik et al. carried out a total extraction using ethyl acetate. The results showed an IC_50_ of 32.86 µg/mL in the PANC-1 line [39] (Table 4 and Figure 5). 

### 3.5. Clinical Trials Carried Out with Secondary Metabolites from Fungi

In addition to the great therapeutic efficacy shown by several compounds, it should be noted that there are currently several extracts and secondary metabolites derived from fungi that are being tested in clinical trials with promising therapeutic activity. Thus, in colorectal cancer, a conjugate called CT-2106 derived from camptothecin and crude extract of camptothecin (genus *Fusarium*) has been developed and tested in combination with the drugs 5-FU and folic acid (NCT00291785), although the definitive results of the phase I/II study have not been published [40]. Another synthetic compound called TNP-470 and derived from fumagillin (*Aspergillus fumigates*) has been approved as an antiangiogenic for the treatment of several types of cancer (breast, renal, prostate, cervical, brain, and Kaposi sarcoma) following a clinical trial (NCT00000763) [41]. Its effect was found to be exerted through the inhibition of the MetAP-2 enzyme. In addition, the administration of this TNP-470 in conjunction with paclitaxel and carboplatin was also tested in another clinical study and may be effective in solid tumors and non-small-cell lung carcinoma [42]. In pancreatic cancer, this compound has been tested together with radiochemotherapy with Gemcitabine for the treatment of locally advanced tumors in a phase II clinical trial. The results of this trial have not yet been published (NCT00038701). 

In addition to these, CKD-732 is a metabolite extracted from the fungus *Aspergillus fumigates* that possesses antiangiogenic activity. It has been tested in phase I trials in combination with the drugs capecitabine and oxaliplatin in patients with metastatic colorectal cancer progressing on irinotecan-based chemotherapy for tolerance and safety [43], as well as in phase II trials to test the optimal treatment dose [44]. Another synthetic derivative compound derived from a fungal metabolite tested in clinical trials was irofulven, an analogue of illudin S (produced by *Omphalotus illudens*) [45]. It is an alkylating agent that has been tested in patients with stage III or IV pancreatic cancer, although the definitive results of the trial were not published, as well as in ovarian and peritoneal cancer (NCT00053365), prostate cancer (NCT03643107) [46], unresectable hepatocellular carcinoma [47], and prostate cancer (NCT03643107). Additionally, this same compound has been tested in combination with the drug oxaliplatin in phase I-II for the treatment of liver cancer (NCT00374660). Finally, plinabulin is another compound derived from the fungus *Aspergillus ustus* that has shown positive results in the treatment of various types of solid tumors and lymphomas [48] (Table 5). 

## 4. Discussion

The first thing to note is that most of the compounds analyzed are derived from the phylum Ascomycota, the rest being derived from Basidiomycota, both forming the subkingdom called Dikarya. They have been described as the major producers of secondary metabolites in the fungal kingdom, both in their sexual (fruiting bodies) and asexual (conidial) phases [49]. In addition, we have observed that the genus Aspergillus is the one that provides the most secondary metabolites with antitumor activity. This genus, in co-culture with other microorganisms such as bacteria, plants, and other fungi, can generate many secondary metabolites, as shown in this study [50]. As can be seen, the different compounds analyzed have been tested in several pancreatic tumor lines, with PANC-1, MIA PaCa-2, and BxPC-3 being the majority throughout the studies. These compounds are usually extracted using organic solvents such as ethyl acetate, ethanol, n-butanol, chloroform, and methanol, although in two studies, the extraction was performed using aqueous solvents [26,33]. The knowledge of which are the most important solvents for the extraction of these bioactive compounds is relevant for the development of future research that attempts to extract compounds of interest from other different species. In view of the results, the great majority of authors use polar organic solvents such as ethyl acetate, since they allow the extraction of water-soluble and hydrophobic molecules, so the variety of compounds obtained is greater [8,51]. Added to the use of these extraction solvents, the high-performance liquid chromatography (HPLC) technique allowed the isolation of specific bioactive molecules from these extracts for their subsequent characterization at the chemical and biological level, being able to obtain pure compounds that possess high in vitro and in vivo antitumor efficacy, as observed in most of the studies [52]. Nevertheless, in four of the studies, the complete extracts of the fungi were evaluated, analyzing the antitumor efficacy of the total internal metabolites possessed by each of the species [32,33,38,39]. 

With respect to their antitumor efficacy, it should be noted that the great majority have their IC_50_ in the micromolar range, making them comparable with many of the traditional chemotherapeutics. Among the most effective, we highlight secalonic acid F (derived from *Aspergillus aculeatus*), with IC_50_ between 2.5 and 3 µM in the lines where it had been tested thanks to its great genotoxic capacity, secoemestrin C, with IC_50_ between 1 and 4 µM in several pancreatic tumor lines, and libertellenone H, derived from a strain of the genus *Eutypella*, which has IC_50_ between 0.67 and 5.53 µM [17,22,34]. Meanwhile, other studies describe that isolated secondary metabolites contribute to the inhibition of the cell’s energy metabolism, resulting in a large difference between their IC_50_ values in low-glucose media and in media with normal energy supplementation. This occurs in two metabolites (physcion and 2-(2′,3-epoxy-1′,3′,5′-heptatrienyl)-6-hydroxy-5-(3-methyl-2-butenyl) benzaldehyde) generated by *Aspergillus* sp. strain 18B-15-3, in the epidithiodiketopiperazine DC1149B generated by *Trichoderma lixii* strain 15G49-1, and in (3S,6S)-3,6-dibenzylpiperazine-2,5-dione generated by *Paecilomyces formosus* strain 17D47-2 [16,23,27]. 

**Figure 6:**
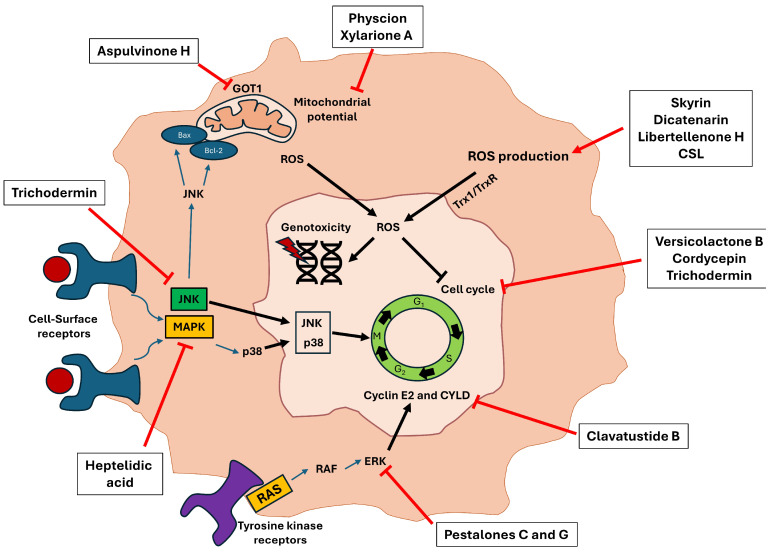
Molecular pathways inhibited by several secondary metabolites reviewed in the studies that ultimately led to the induction of cell apoptosis. The different bioactive compounds can perform their cytotoxic activity through different routes such as inducing intracellular ROS production, altering the mitochondrial membrane potential, or inhibiting proteins involved in tumor malignancy and proliferation, such as JNK, MAPK, and ERK. All these pathways lead to the production of genotoxicity and cycle arrest, producing an inhibition of tumor growth.

Reviewing the experiments carried out in the studies, it should be noted that all of them analyze growth inhibition through various techniques, such as Sulforhodamine B, MTT, MTS, or CCK-8. Although, in many of the studies, the induced cell death pathway is not analyzed, in more than half (14/27), death is generated by apoptosis, being analyzed by flow cytometry and Western blot. In others, arrests occur at different points in the cell cycle. Thus, Yan et al. and Qi et al. described that the compounds aspulvinone H, (+)-3′,3′-di-(dimethylallyl)-butyrolactone II and versicolactone B (derived from the fungus *Aspergillus terreus*) produced cycle arrest in the S phase, the same effect carried out with cordycepin (*Cordyceps militaris*) [14,15]. Others carried out this arrest in the G1/S phase, producing a deregulation on two cyclins in charge of controlling the cell cycle (cyclin E2 and CYLD) [18]. Finally, quiescence phase arrest (G0/G1) was characterized by the compounds xylarione A, (−) 5-methylmellein, trichodermin, as well as the complete aqueous extract derived from the fungus *Agaricus blazei* Murrill [30,33,37]. Furthermore, in many of the studies in which apoptosis and cycle arrest were generated, the cause of these phenomena was due to the induction of intracellular ROS [14,21,26,34] (Figure 6).

Due to the high antitumor efficacy shown by these compounds in vitro, in some of the included studies, mouse trials were also carried out. Thus, Shi et al. carried out a xenograft tumor model using the BxPC-3 line, which showed the capacity of the isolated compound dicitronone G to possess antiangiogenic activity and inhibition of tumor growth [20]. Using this same model, Li et al. analyzed the antitumor efficacy of cordycepin, observing a strong inhibition of tumor growth through inhibition of the FGFR/Ras/ERK pathway and deregulation of Ki67 [25]. Meanwhile, other studies were carried out using models generated from cell lines SW1990, SUIT-2, and MIA PaCa-2, where the antitumor efficacy of the compounds studied was observed in comparison to chemotherapeutic drugs such as 5-FU [14,19,30].

In addition to the low number of clinical studies shown above (Table 5), it is also observed that these are in early stages (I or II), so it is necessary to find new compounds that may have an interesting therapeutic potential against different types of cancer. In this context, the number of compounds that can be derived from fungi is very high, so it is necessary to increase the capacity to extract these molecules and to test them in vitro and in vivo. A limitation of this study may be the fact that none of the studies have tested the combination of bioactive compounds/extracts together with clinically used chemotherapeutics. Since many of these act by inhibiting specific signaling pathways involved in cancer development, such as p38, ER, or JNK [19,22,35], their combination with chemotherapeutic drugs used in PC (such as Gemcitabine) could be interesting. This would allow us to reduce the administered dose of the drug, producing a similar effect and decreasing toxicity in the case of a synergistic effect. In natural compounds derived from plants, such as polyphenols, flavonoids, or terpenes, synergies have been observed with several drugs in colon cancer (5-FU, irinotecan, or oxaliplatin), so the performance of this type of study would be of great interest [53].

Future perspectives in this field of study are based on the use of new solvents that allow the isolation of new bioactive molecules from nature, avoiding the toxicity, volatility, and flammability of the solvents currently in use. In this context, deep eutectic solvents (DESs) and natural deep eutectic solvents (NaDESs) can be interesting alternatives to avoid all these problems. Among them, we can highlight combinations of choline chloride with different acids (lactic, malic, and citric), combinations of sugars (fructose with diluted sucrose), sugars together with polyols, or even mixtures of sugars with organic acids (fructose with lactic acid, for example). This type of solvent would allow the use of specific combinations for the extraction of compounds of interest, improving their stability and with low volatility and toxicity in comparison with the different solvents mostly used in this review (mainly ethyl acetate and ethanol) [54]. Furthermore, it would be interesting to encapsulate these bioactive molecules in nanoparticles to try to increase their cytotoxic effect in PC, decreasing the possible systemic toxicity and increasing the availability of the drug in the tumor through the enhanced permeability and retention (EPR) effect [55]. Due to the general low bioavailability of these compounds when administered to humans, nanotechnology allows the stabilization of these metabolites and prevents their degradation in the blood, thus increasing the half-life time and their potential therapeutic efficacy. Added to this, these nanoparticles allow us to evade several routes of compound detoxification and, therefore, of drug resistance that tumor cells possess. Therefore, they are also an interesting treatment route for the combination of these secondary compounds together with traditional chemotherapeutics, which could generate pharmacological synergies that could be interesting as a therapy against PC [56]. In addition, fungi are useful for the “green synthesis” of magnetic nanoparticles. For this purpose, the mixing of metal salts together with fungi is usually performed to employ their metabolism with reducing power, realizing the reduction of metal salts, and allowing the synthesis of these nanoformulations [57,58]. Despite this, none of the compounds included in this systematic review have been studied while encapsulated in nanoparticles, so it is a future therapeutic option that should be studied and that could be interesting in some of the most interesting compounds seen previously, such as secalonic acid F, secoemestrin C, and libertellenone H.

## 5. Conclusions

In conclusion, several bioactive compounds of great interest derived from fungi of the phyla Ascomycota and Basidiomycota that possess an interesting cytotoxic effect on different PC lines have been identified in 27 studies included in this systematic review. Some of the compounds characterized during the last 10 years with the best cytotoxic activity are secalonic acid F, secoemestrin C, and libertellenone H, which had IC_50_ values in the µM range and, in many cases, were more effective than the chemotherapy currently used in the clinic. Most of the compounds included carried out their antitumor action through the induction of apoptosis via cell cycle arrest, induction of genotoxicity, and induction of intracellular ROS. Additionally, the most employed extraction method for these was the use of an organic solvent such as ethyl acetate (with great capacity to dissolve compounds due to its polarity), coupled with HPLC isolation. The results obtained in in vivo assays with mice showed the efficacy of these compounds in several xenograft models generated from PC lines, showing their ability to inhibit tumor growth in a concentration-dependent manner. Nevertheless, the small number of clinical trials that have been conducted with fungal-derived secondary metabolites so far highlights the need for further research into the discovery of new compounds that will improve the therapeutic efficacy of this type of cancer.

## Figures and Tables

**Table 1:** Factors valued for developing quality assessment.

Score (Each Item)	Factors Considered
3 points	The article has been reviewed through peer-review
	The IC_50_ values of the described compounds are specified.
2 points	The extraction/synthesis process is correctly explained
	The compound responsible for the antitumor activity is identified
	The results represented have error bars (SD)
	The species from which the metabolite is derived is indicated
1 point	Methodology is detailed
	At least one non-tumor line is included
	The metabolic pathway by which the compound acts is analyzed
	It is tested on 2 or more cell lines (tumor type analyzed)
	It is tested on cell lines from other types of tumors
	An in vivo test of the compound has been carried out

**Table 2:** Summary of the main characteristics of the studies carried out with secondary compounds derived from fungi of the order Eurotiales.

Ref.	Fungi	Compound (Extraction and Isolation Method)	IC_50_ (Hours of Treatment)	Pathway	In Vitro Results	In Vivo Results
[11]	*Aspergillus terreus*	(−)-asperteretone E (EA and HPLC)	9.5, 9.8, 11.7 and >40 µM in AsPC-1, PANC-1, SW1990 and SW480, respectively (48 h)	NA	Cell growth inhibition	NA
		(+)-asperteretone E (EA and HPLC)	9.9, 15.6, 10.3 and >40 µM in AsPC-1, PANC-1, SW1990 and SW480, respectively (48 h)			
		Compound 6 (EA and HPLC)	5.6, 9.1, 1.2 and >40 µM in AsPC-1, PANC-1, SW1990 and SW480, respectively (48 h)			
[14]		Aspulvinone H (EtOH and HPLC)	Between 6.32-10.47 µM in SW1990, PANC-1 and AsPC-1 cell lines (48 h)	GOT1	Increased ROS stress, apoptosis induction, S-phase cell cycle stop and decreased cell migration	Dose-dependent tumor growth inhibition in SW1990 xenograft model
[15]		(+)-3′,3′-di-(dimethylallyl)-butyrolactone II (EtOH and HPLC)	5.3 µM in PANC-1 cells (24 h)	NA	Cell growth inhibition, apoptosis induction and S phase cell cycle stop	NA
		Versicolactone B (EtOH and HPLC)	9.4 µM in PANC-1 cells (24 h)			
[16]	*Aspergillus* sp. 18B-15-3	Physcion (ACE and HPLC)	6 µM and 1.01 mM in glucose-deficient and normal medium, respectively, in PANC-1 (12 h)	NA	Cell growth inhibition and decreased mitochondrial membrane potential	NA
		2-(2′,3-epoxy-1′,3′,5′-heptatrienyl)-6-hydroxy-5-(3-methyl-2-butenyl) benzaldehyde (ACE and HPLC)	1.7 µM and 0.86 mM in glucose-deficient and normal medium, respectively, in PANC-1 (12 h)			
[17]	*Aspergillus aculeatus*	Secalonic acid F	2.5 and 3 µM in BxPC-3 and SU86.86 cells, respectively (72 h)	NA	Cell growth inhibition and genotoxicity induction	NA
[18]	*Aspergillus clavatus* C2WU	Clavatustide B (EA/n-But extraction)	Between 30–40 (48 h) and 20–30 (72 h) μg/mL in PANC-1 cells	Cyclin E2 and CYLD	Cell growth inhibition and G1/S phase cell cycle stop	NA
[19]	*Aspergillus oryzae*	Heptelidic acid (HPLC on supernatant)	NA	p38/MAPK	Cell growth inhibition and apoptosis induction	Tumor growth inhibition via p38/MAPK downregulation in SUIT-2 xenograft model
[20]	*Penicillium* sp. GGF 16-1-2	Dicitrinone G (EA and HPLC)	NA	NLRP3/ IL-18	Angiogenesis and cell growth inhibition in HUVEC cells	Angiogenesis and tumor growth inhibition in BX-PC3 xenograft model
[21]	*Penicillium pinophilum*	Skyrin (EA and HPLC)	27 μg/mL in MIA PaCa-2 cells (48 h)	NA	Cell growth inhibition, apoptosis induction, ROS induction and colony formation inhibition	NA
		Dicatenarin (EA and HPLC)	12 μg/mL in MIA PaCa-2 cells (48 h)			
[22]	*Emericella striata* and *Emericella quadrilineata*	Secoemestrin C	Between 1–4 µM in AsPC-1, BxPC-3, MIA PaCa-2, PANC-1, SU86.86 and SW1990 cell lines (48 h)	ER/YAP	Cell growth and colony formation inhibition, induction of apoptosis and disordered lipid biosynthesis	NA
[23]	*Paecilomyces formosus* 17D47-2	(3S,6S)-3,6-dibenzylpiperazine-2,5-dione (NA)	28 µM and >1 mM in glucose-deficient and normal medium, respectively, in PANC-1 (time undefined)	NA	Cell growth inhibition and increased oxygen consumption	NA

ACE (acetone); EA (ethyl acetate); EtOH (ethanol); HPLC (high-performance liquid chromatography); NA (non-accessible); n-But (n-buthanol); ROS (reactive oxygen species).

**Table 3:** Summary of the main characteristics of the studies carried out with secondary compounds derived from fungi of the order Hypocreales.

Ref.	Fungi	Compound (Extraction and Isolation Method)	IC_50_ (Hours of Treatment)	Pathway	In Vitro Results	In Vivo Results
[24]	*Isaria* sp. RD055140	Beauvericin (EA and HPLC)	4.8 µM in PANC-1 (48 h)	NA	Cell growth inhibition, decreased cell migration and regulation of EMT-related gene expression	NA
[25]	*Cordyceps militaris*	Cordycepin (NA)	38.85, 72.99, 150.1, 213.1 and 349.3 µM in BxPC-3, CFPAC-1, AsPC-1, PANC-1 and SW1990 (72 h)	FGFR2	Cell growth and colony formation inhibition, decreased cell migration, induction of apoptosis and S phase cell cycle stop	Tumor growth inhibition via FGFR/Ras/ERK and downregulation of Ki67 expression in BX-PC3 xenograft model
[26]	*Cephalosporium curvulum*	Cephalosporium curvulum lectin (CSL) (Sodium phosphate buffer and HPLC)	Between 2.5 and 5 µg/mL in PANC-1 cells (72 h)	NA	Cell growth inhibition, apoptosis induction and ROS induction	NA
[27]	*Trichoderma lixii* 15G49-1	Epidithiodiketopiperazine DC1149B (ACE/MeOH/EA (4:2:1) and HPLC)	0.02 µM and 710 mM in glucose-deficient and normal medium, respectively, in PANC-1 (12 h)	ER	Cell growth inhibition and decreased oxygen consumption	NA
[28]	*Purpureocillium lilacinum* 40-H-28	Leucinostatin A (ACE, CL and MeOH and HPLC)	0.05 µg/mL in PANC-1 (72 h)	NA	Cell growth inhibition	NA
		Leucinostatin Y (ACE, CL and MeOH and HPLC)	4.1 µg/mL in PANC-1 (72 h)			
[29]	*Fusarium solani*	Fusaproliferin (EA and HPLC)	0.13 and 0.76 µM in MIA PaCa-2, BxPC-3 cells, respectively (96 h)	NA	Cell growth inhibition	NA
[30]	*Nalanthamala psidii*	Trichodermin (NA)	0.8, 1.2, 1.4 and 18.8 µM in MIA PaCa-2, BxPC-3, HPAC and hTERT-HPNE cells (48 h)	JNK	Cell growth and colony formation inhibition, apoptosis and genotoxicity induction and G0/G1 phase cell cycle stop	Tumor growth inhibition and apoptosis induction in MIA PaCa-2 xenograft model

ACE (acetone); CL (chloroform); EA (ethyl acetate); EMT (epithelial–mesenchymal transition); HPLC (high-performance liquid chromatography); MeOH (methanol); NA (non-accessible); ROS (reactive oxygen species).

**Table 4:** Summary of the main characteristics of the studies carried out with secondary compounds derived from fungi of other orders.

Ref.	Fungi	Compound (Extraction and Isolation Method)	IC_50_ (Hours of Treatment)	Pathway	In Vitro Results	In Vivo Results
[31]	*Cyathus striatus*	Striatal C (EA and HPLC)	2.5–5 µg/mL (8 and 12 h) and 0–2.5 µg/mL (24 h) in HPAF-II and PL45 cell lines	NA	Cell growth inhibition	NA
[32]	*Calocybe indica*	Unknown (EtOH)	245 and 332 µg/mL in PANC-1 and MIA PaCa-2 cells, respectively (24 h)	NA	Cell growth inhibition, LDH cell leakage, decreased cell migration and apoptosis induction	NA
[33]	*Agaricus blazei* Murrill	Unknown (aqueous extraction)	NA	NA	Cell growth inhibition, apoptosis induction and G0/G1 phase cell cycle arrest	NA
[34]	*Eutypella* sp. D-1	Libertellenone H (EA and HPLC)	0.67, 2.78, 3.21 and 5.53 µM in SW1990, AsPC-1, PANC-1 and BxPC-3, respectively (48 h)	Trx1/TrxR system	Cell growth inhibition, apoptosis induction and ROS induction	NA
[35]	*Pestalotiopsis neglecta* LK29	Pestalone C (EA/MeOH and HPLC)	7.6 µM in PANC-1 cells (72 h)	MEK/ERK	Cell growth and colony formation inhibition and induction of apoptosis	NA
		Pestalone E (EA/MeOH and HPLC)	7.2 µM in PANC-1 cells (72 h)			
[36]	*Dothiora* sp.	Hormonemate E (ACE and HPLC)	36.4 µM in MIA PaCa-2 (72 h)	NA	Cell growth inhibition	NA
[37]	*Xylaria psidii*	Xylarione A (EA and HPLC)	16 µM in MIA PaCa-2 cells (48 h)	NA	Cell growth inhibition, apoptosis induction, G0/G1 phase cell cycle stop and decreased mitochondrial membrane potential	NA
		(−) 5-methylmellein (EA and HPLC)	19 µM in MIA PaCa-2 cells (48 h)			
[38]	*Antrodia camphorata*	Unknown (EA and EtOH)	2.49 (EE) and 2.36 (EA) μg/mL in BxPC-3 cells (48 h)	NA	Cell growth inhibition, apoptosis induction, G2/M phase cell cycle stop and genotoxicity	NA
[39]	*Colletotrichum gloeosporioides*	Unknown (EA)	32.86 μg/mL in PANC-1 cells (48 h)	NA	Cell growth inhibition	NA

ACE (acetone); EA (ethyl acetate); EtOH (ethanol); HPLC (high-performance liquid chromatography); LDH (lactate dehydrogenase); MeOH (methanol); NA (non-accessible); ROS (reactive oxygen species).

**Table 5:** Clinical trials that have tested mushroom-derived bioactive compounds in different types of cancer.

Compound	Fungi	Combined Therapy	Type of Cancer	Results	Last Phase (ID Number)
CT-2106	*Fusarium* sp.	5-FU and folic acid	Colorectal cancer	A total of 26 patients included with 6 doses on average. The most frequent tumor was melanoma. Most toxicities were hematological, with the maximum tolerated dose being 25 mg/m^2^ and being better than unencapsulated campothecin.	Phase I/II (NCT00291785)
TNP-470	*Aspergillus fumigates*	Gemcitabine chemoradiation	Locally advanced pancreatic cancer	NA	Phase II (NCT00038701)
		Not combined	AIDS-Associated Kaposi’s Sarcoma	A total of 42 patients with 7 escalating dose levels. Weekly administration of the drug is tolerated at the highest dose administered (70 mg/m^2^). Tumor response was observed in several cases and at various doses tested.	Phase I (NCT00000763)
CKD-732	*Aspergillus fumigates*	Capecitabine and oxaliplatin	Metastatic colorectal cancer	A total of 9 patients that progressed on irinotecan-based chemotherapy included; 2, 5, and 10 mg/m^2^/d were administered twice weekly for 2 weeks, in combination with oxaliplatin and capecitabine. The clinical recommended dose was 5 mg/m^2^/d in combination with XELOX, with adverse events including insomnia and fatigue.	Phase Ib (NA)
Irofulven (illudin S analogue)	*Omphalotus illudens*	Not combined	Recurrent or persistent platinum- sensitive ovarian or primary peritoneal cancer	A total of 55 patients were enrolled, 7 with partial response and 30 with disease stabilization. Disease-free progression and median survival were 6.4 and 22.1 months, respectively. Good tolerance but low activity as single therapy.	Phase II (NCT00053365)
		Prednisolone	AR- and docetaxel-sensitive metastatic castration-resistant prostate cancer	NA (active at this moment)	Phase II (NCT03643107)
		Oxaliplatin	Locally advanced liver cancer	A total of 63 patients were enrolled, but results are not accessible.	Phase I-II (NCT00374660)

AR: androgen receptor; 5-FU: 5-fluorouracil; NA: non-accesible.

## Data Availability

This systematic review was registered in the OSF database (osf.io) on 4 December 2023, and can be accessed through the following link: https://doi.org/10.17605/OSF.IO/6Y5JQ (accessed on 10 July 2024).
